# Correlation of Frontal Sinus Development With Skeletal Maturation of Cervical Vertebrae and Dental Calcification Stages of Permanent Teeth: A Cross-Sectional Study

**DOI:** 10.7759/cureus.102486

**Published:** 2026-01-28

**Authors:** Aswathy Sudarsanan, Elaine S Barretto, Dinesh F Swamy

**Affiliations:** 1 Department of Pediatric and Preventive Dentistry, Goa Dental College and Hospital, Bambolim, IND

**Keywords:** cervical vertebrae, dental calcification, frontal sinus, growth and development, skeletal maturity

## Abstract

Introduction: Skeletal maturity evaluation plays a critical role in orthodontic treatment planning. While cervical vertebral maturation index (CVMI) and dental calcification stages are commonly used, the potential of frontal sinus dimensions as an additional indicator has gained attention. This study evaluated the correlation of frontal sinus development with skeletal maturation of cervical vertebrae and calcification stages of permanent teeth.

Materials and methods: The sample consisted of 60 subjects, with a mean age of 13 ± 3.4 (range 8-20) years. Standardized pre-treatment lateral cephalograms were analyzed. Six groups representing different phases of CVMI were created from the entire sample (five males and five females in each group). Frontal sinus maturation was assessed on the same lateral cephalograms. Calcification stages of permanent canines and permanent second molars were assessed according to Demirjian’s method, from panoramic radiographs of corresponding subjects taken on the same day. Correlations between the maturation of the frontal sinus with CVMI and calcification stages of permanent canines and permanent second molars were assessed using statistical analysis.

Results: Frontal sinus height (FS-H) and depth (FS-D) demonstrated statistically significant positive correlations with advancing CVMI stages and moderate positive correlations with calcification stages of permanent canines and second molars, particularly in males, whereas frontal sinus index (FSI) consistently showed no significant correlation across all groups. Post hoc analyses further confirmed significant differences in sinus dimensions between early and late CVMI stages.

Conclusions: This study concludes that FS-H and FS-D are moderately reliable indicators of skeletal and dental maturity, aligning closely with CVMI and dental calcification stages, particularly in males, and thereby improving treatment planning.

## Introduction

Skeletal maturity assessment is crucial in pediatric dentistry and orthodontics to guide treatment timing. While cervical vertebral maturation index (CVMI) and hand-wrist radiographs are standard, they involve additional radiation and observer variability. Dental calcification stages, assessed via panoramic radiographs using Demirjian’s or Nolla’s methods, offer a non-invasive alternative [1-4]. Frontal sinus development, visible on lateral cephalograms (LCs), has also shown potential correlation with skeletal growth [5-12]. However, its relationship with dental maturity remains unexplored.

Given the well-documented relationship between dental calcification and skeletal maturation, integrating frontal sinus development with dental and cervical vertebral assessments may enhance the accuracy of evaluation, as well as offer a more holistic framework for skeletal maturity assessment. Such an integrated approach may improve diagnostic precision in orthodontics, forensic science, and growth-related research.

The aim of the study was to establish the correlation between frontal sinus dimensions, CVMI stages, and dental calcification stages of permanent teeth, in individuals aged 8-20 years, to support more accurate, growth-based treatment planning.

The primary objective was to assess the relationship between frontal sinus dimensions, CVMI stages, and dental calcification stages of permanent teeth. The secondary objectives were to assess the same separately for males and females.

## Materials and methods

This cross-sectional study was conducted at Goa Dental College and Hospital, Bambolim, Goa, from December 2024 to March 2025, using LCs and orthopantomograms (OPGs). Ethical clearance was obtained from the Institutional Review Board, and the study was registered with the Open Science Framework (osf.io/6abn5).

The study included anonymized, high-quality LCs and OPGs taken on the same day from individuals aged eight to 20 years (males and females) with normal growth and development. Subjects were excluded if they had any systemic conditions or diseases affecting skeletal or dental tissues, frontal sinus pathologies, a history of growth modification or orthodontic treatment, trauma or surgery, or congenitally missing teeth.

Sample size calculation based on a study done by Mahmood et al. in 2016 [10], yielding 43 subjects. To allow equal distribution across six growth phases as per Baccetti et al.'s [13]CVMI method, 17 additional samples were added, making a total of 60.

Formula used for sample size calculation:

$\text{Total sample size} = N = \left[\frac{Z_{\alpha} + Z_{\beta}}{C}\right]^2 + 3 = \left[\frac{1.96 + 0.8416}{0.4433}\right]^2 + 3 = 43$

$C = 0.5 \times \ln\left(\frac{1 + r}{1 - r}\right) = 0.5 \times \ln\left(\frac{1 + 0.414}{1 - 0.414}\right) = 0.4433$

The expected correlation coefficient, r = 0.414 (based on the study done by Mahmood et al. [10]).

**Figure 1:**
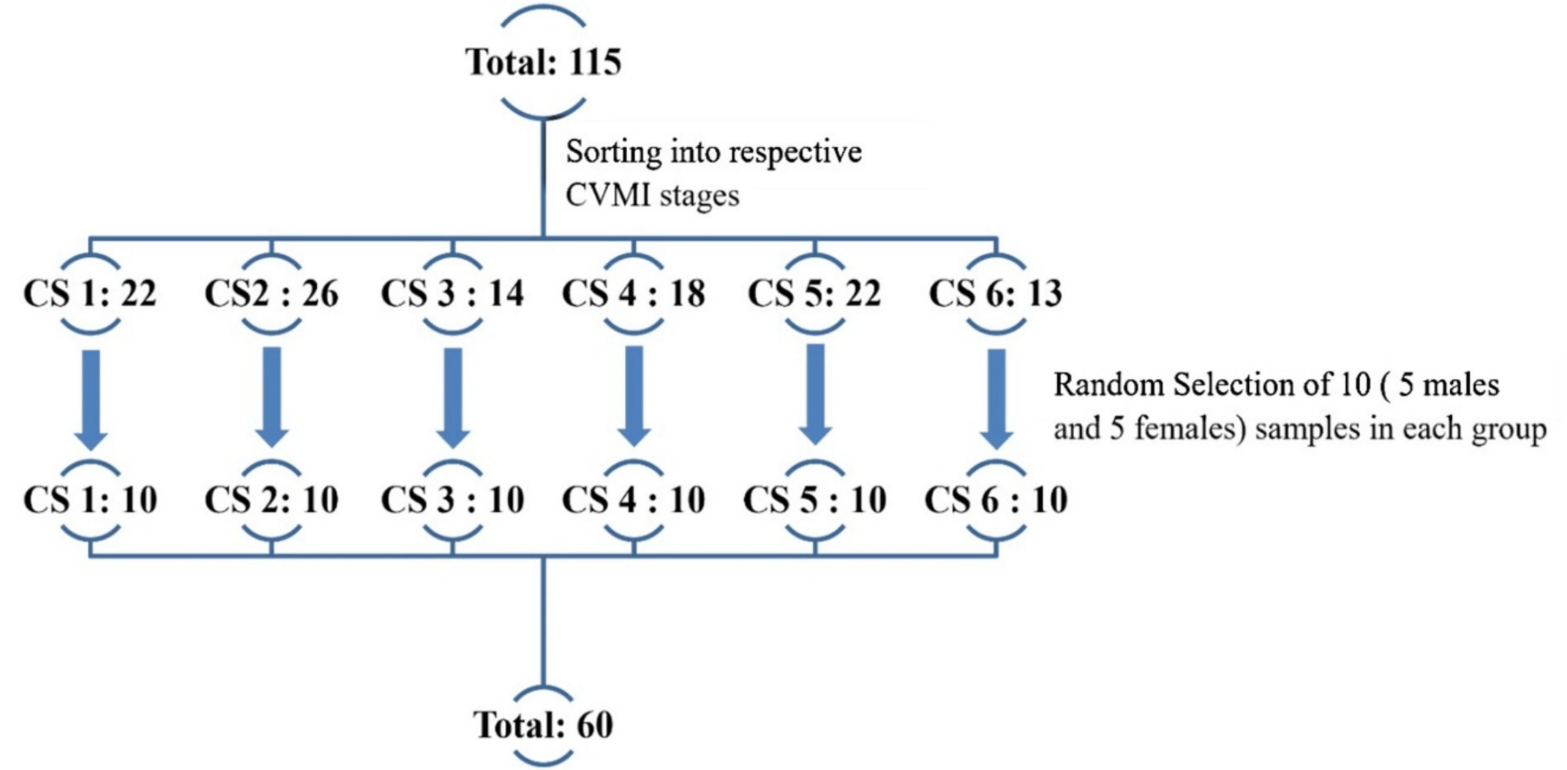
Diagrammatic representation of division of samples. CVMI stages were assessed using the method by Baccetti et al. in 2005 [13]. Sample size calculation was done based on a study done by Mahmood et al. in 2016 [10]. CVMI: cervical vertebral maturation index

Initially, 115 LCs of individuals aged 8-20 years were randomly selected, stratified by sex, and anonymized using masking tape for blinding. These were evaluated and classified into six CVMI stages by one examiner, using Baccetti et al.’s [13] six-stage method as shown in Figure 1. From each stage, five male and five female radiographs were randomly selected via lottery, ensuring unbiased representation.

Frontal sinus measurements were performed using Ertuk’s method [11,14]. LCs were aligned to the Sella-Nasion (SN) plane. Frontal sinus height (FS-H) was determined by drawing a vertical line between the highest and lowest points of the sinus. Frontal sinus depth (FS-D) was obtained by a line connecting the most anterior and posterior points, which is perpendicular to the vertical line of FS-H. To reduce magnification error, the frontal sinus index (FSI) was calculated as a ratio of FS-H to FS-D and recorded individually for each subject.

On the same day, OPGs were used to assess calcification stages of the permanent canine and second molar using Demirjian’s index (DI), which categorizes tooth development into eight stages (A to H) based on crown and root morphology [1]. All skeletal and dental assessments were performed by a calibrated examiner (intra-observer reliability: Cohen’s kappa statistic, κ = 0.93), and each parameter was independently cross-verified by a blinded experienced orthodontist.

The data were statistically analyzed using IBM SPSS Statistics for Windows, Version 23 (Released 2016; IBM Corp., Armonk, New York, United States). Descriptive statistics, including median values and interquartile ranges, were used to summarize frontal sinus measurements across different CVMI stages. Differences in frontal sinus measurements among CVMI stages were assessed using the Kruskal-Wallis test, followed by Dunn’s post-hoc test for pairwise comparisons. Spearman’s rank correlation coefficient was applied to evaluate the association between frontal sinus development, CVMI stages, and dental calcification stages of permanent teeth. Additionally, Spearman correlation analysis was performed separately for males and females to identify any sex-related differences. This analytical approach enabled a clear and reliable assessment of developmental relationships among the studied variables.

## Results

**Figure 2:**
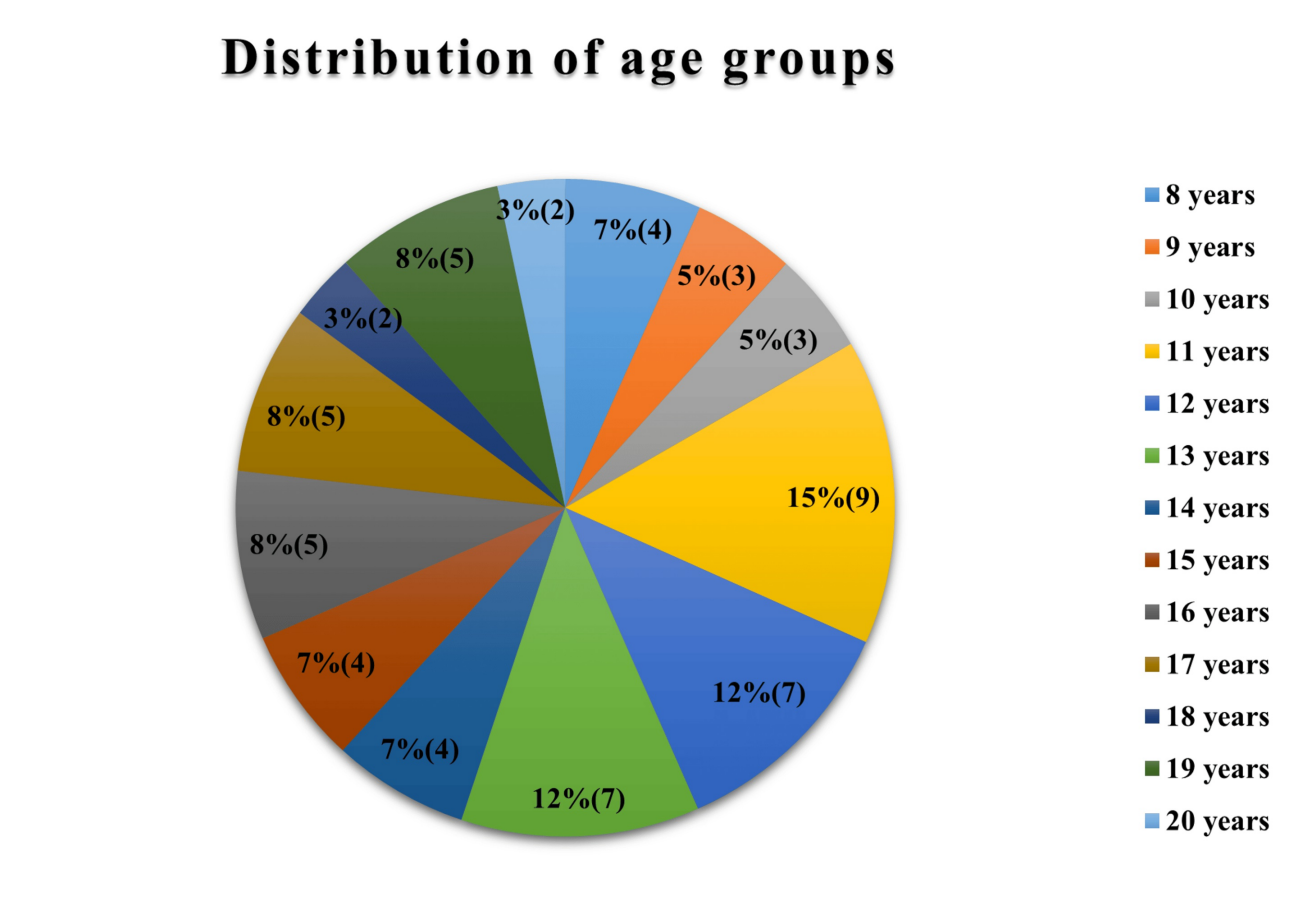
Age-wise distribution of the subjects.

The study population consisted of a total sample of 60 (30 males and 30 females) of ages ranging from eight to 20 years old, with a mean age of 13 ± 3.4 years. Figure 2 shows the demographic details of the study subjects.

Using the Kruskal-Wallis test, statistically significant differences were observed in both FS-H (p = 0.001) and FS-D (p = 0.008) across CVMI stages, whereas FSI did not demonstrate a significant variation (p = 0.395). Table 1 shows the comparison of FS-H, FS-D, and the index with CVMI stages.

**Table 1:** Comparison of frontal sinus height, depth, and index across CVMI stages using the Kruskal-Wallis test. p < 0.05 indicates statistical significance; p < 0.001 indicates high statistical significance. CVMI stages were assessed using the method described by Baccetti et al. (2005) [13]. Frontal sinus dimensions were measured, and the frontal sinus index was calculated based on the study by Alijani et al. (2020) [11], which was derived from the method described by Ertürk (1968) for frontal sinus measurements [14]. CVMI: cervical vertebral maturation index

CVMI stage	Frontal sinus	Median	Interquartile range	Kruskal-Wallis H	p-value
I	Height	24.8	21.5-25.0	20.600	0.001
II		26.3	17.0-27.5		
III		31.0	29.0-34.0		
IV		26.0	25.0-32.0		
V		32.5	26.0-35.0		
VI		34.0	31.0-38.0		
I	Depth	9	8-9.5	15.723	0.008
II		10	09-12		
III		11.5	9-13		
IV		11.5	9-13.5		
V		13	11-15		
VI		13.5	9-15		
I	Index	2.72	2.25-3.3	5.178	0.395
II		2.36	1.83-2.54		
III		2.72	2.38-3.00		
IV		2.52	2.08-2.89		
V		2.27	2.00-2.73		
VI		2.76	2.10-3.33		

Post hoc analysis (Dunn’s test) revealed that FS-H and FS-D values were notably higher in advanced CVMI stages, particularly between stages I vs. VI and II vs. VI (p < 0.05). Table 2 shows post hoc multiple comparisons of FS-H and FS-D among CVMI stages using Dunn’s test.

**Table 2:** Post hoc multiple comparisons of FS-H and FS-D among CVMI stages using Dunn’s test. p < 0.05 indicates statistical significance; p < 0.001 indicates high statistical significance. CVMI stages were assessed using the method described by Baccetti et al. (2005) [13]. Frontal sinus dimensions were measured based on the study by Alijani et al. (2020) [11], which was derived from the method described by Ertürk (1968) for frontal sinus measurements [14]. CVMI: cervical vertebral maturation index; FS-H: frontal sinus height; FS-D: frontal sinus depth

Multiple comparisons	Dunn’s test statistic for FS-H	FS-H p-value	Dunn’s test statistic for FS-D	FS-D p-value
I vs. II	-3.900	1.000	-10.400	1.000
I vs. III	-18.950	1.000	-14.450	0.940
I vs. IV	-12.600	0.227	-17.350	0.381
I vs. V	-21.550	0.086	-27.250	0.036
I vs. VI	-29.700	0.002	-23.550	0.007
II vs. III	-15.050	1.000	-4.050	1.000
II vs. IV	-8.700	0.805	-6.950	1.000
II vs. V	-17.650	0.355	-16.850	1.000
II vs. VI	-25.800	0.014	-13.150	0.449
III vs. IV	-6.350	1.000	-2.900	1.000
III vs. V	-2.600	1.000	-12.800	1.000
III vs. VI	-10.750	0.426	-9.100	1.000
IV vs. V	-8.950	1.000	-9.900	1.000
IV vs. VI	-17.100	1.000	-6.200	1.000
V vs. VI	-8.150	1.000	-3.700	1.000

**Figure 3:**
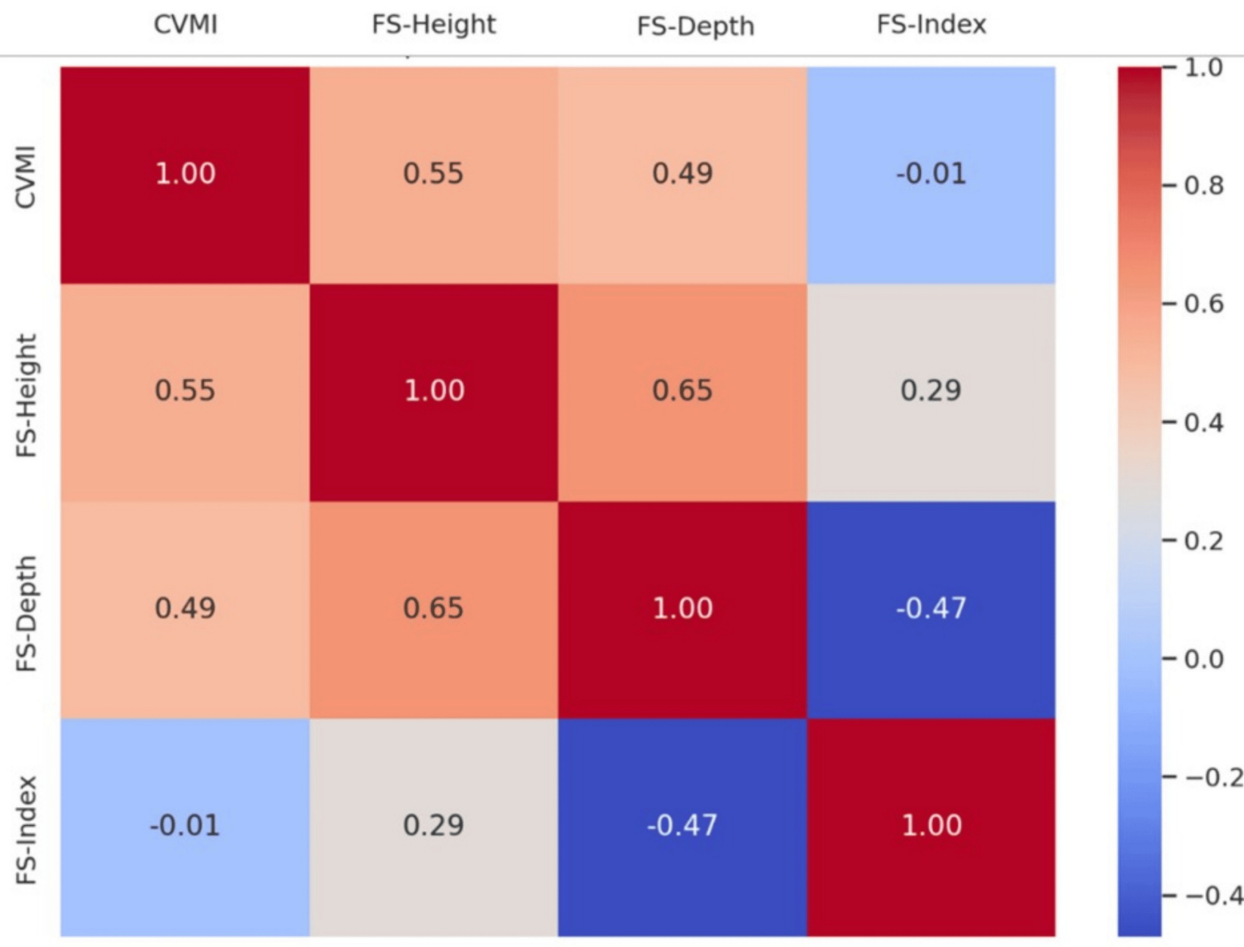
Spearman’s correlation heatmap illustrating the relationships among CVMI, FS-H, FS-D and FSI. CVMI stages were assessed using the method described by Baccetti et al. (2005) [13]. Frontal sinus dimensions were measured, and the frontal sinus index (FSI) was calculated based on the study by Alijani et al. (2020) [11], which was derived from the method described by Ertürk (1968) for frontal sinus measurements [14]. CVMI: cervical vertebral maturation index; FS-H: frontal sinus height; FS-D: frontal sinus depth

Correlation analysis using Spearman’s coefficient showed a moderate positive relationship between CVMI stage and both FS-H (r = 0.550, p < 0.001) and FS-D (r = 0.485, p < 0.001). However, FSI showed no meaningful correlation with CVMI (r = -0.005, p = 0.973). FS-H and FS-D were significantly correlated with each other (r = 0.647, p < 0.001), while FS-D and FSI showed an inverse relationship (r = -0.466, p < 0.001). Figure 3 depicts the correlation heatmap showing the relationships between CVMI, FS-H, FS-D, and FSI.

When frontal sinus dimensions were compared with the CS of permanent teeth, FS-H and FS-D demonstrated statistically significant moderate correlations with CS of permanent canines and second molars (r values ranging from 0.403 to 0.467; p < 0.01). FSI did not show a statistically significant relationship with dental calcification in any of the assessed teeth. The Spearman’s correlation coefficient between CS of permanent teeth and frontal sinus measurements is depicted in Table 3.

**Table 3:** Spearman’s correlation coefficients between calcification stages (CS) of permanent teeth and frontal sinus measurements for all samples. p < 0.05 indicates statistical significance; p < 0.001 indicates high statistical significance. r = 0.20-0.399, low correlation; r = 0.40-0.599, moderate correlation; r = 0.60-0.799, high correlation. * Correlation is significant at the 0.05 level (two-tailed); ** Correlation is significant at the 0.01 level (two-tailed). Dental calcification stages were assessed using the method described by Demirjian et al. (1973) [1]. Frontal sinus dimensions were measured, and the frontal sinus index (FSI) was calculated based on the study by Alijani et al. (2020) [11], which was derived from the method described by Ertürk (1968) for frontal sinus measurements [14]. FS-H: frontal sinus height; FS-D: frontal sinus depth

CS of the tooth	Spearman’s correlation coefficient and p-value	FS-H (mm)	FS-D (mm)	FSI = FS-H/FS-D
CS 13	Correlation coefficient	0.451^**^	0.415^**^	0.034
	p-value	0.000	0.001	0.799
CS 23	Correlation coefficient	0.467^**^	0.419^**^	0.032
	p-value	0.000	0.001	0.806
CS 33	Correlation coefficient	0.451^**^	0.374^**^	0.046
	p-value	0.000	0.003	0.725
CS 43	Correlation coefficient	0.462^**^	0.370^**^	0.081
	p-value	0.000	0.004	0.540
CS 17	Correlation coefficient	0.411^**^	0.401^**^	-0.025
	p-value	0.001	0.002	0.851
CS 27	Correlation coefficient	0.418^**^	0.413^**^	-0.022
	p-value	0.001	0.001	0.869
CS 37	Correlation coefficient	0.410^**^	0.393^**^	-0.008
	p-value	0.001	0.002	0.949
CS 47	Correlation coefficient	0.403^**^	0.376^**^	-0.001
	p-value	0.001	0.003	0.992

Gender-wise correlation analysis revealed that both FS-H and FS-D had a stronger association with CVMI stages among males (r = 0.554 and 0.612, respectively) compared to females (r = 0.552 and 0.399). Table 4 shows gender-wise correlation analysis of FS-H, FS-D, and FSI with CVMI stages among males and females.

**Table 4:** Gender-wise Spearman’s correlation analysis of FS-H, FS-D, and FSI with CVMI stages among males and females. p < 0.05 indicates statistical significance; p < 0.001 indicates high statistical significance. r = 0.20-0.399, low correlation; r = 0.40-0.599, moderate correlation; r = 0.60-0.799, high correlation. * Correlation is significant at the 0.05 level (two-tailed); ** Correlation is significant at the 0.01 level (two-tailed). CVMI stages were assessed using the method described by Baccetti et al. (2005) [13]. Frontal sinus dimensions were measured, and the frontal sinus index (FSI) was calculated based on the study by Alijani et al. (2020) [11], which was derived from the method described by Ertürk (1968) for frontal sinus measurements [14]. CVMI: cervical vertebral maturation index; FS-H: frontal sinus height; FS-D: frontal sinus depth

CVMI stage and sex	Spearman’s correlation coefficient and p-value	FS-H (mm)	FS-D (mm)	FSI = FS-H/FS-D
CVMI stage in males	Correlation coefficient	0.554^**^	0.612^**^	-0.157
	p-value	0.001	0.000	0.408
CVMI stage in females	Correlation coefficient	0.552^**^	0.399^*^	0.228
	p-value	0.002	0.029	0.226

The correlation between sinus dimensions and tooth calcification was more consistent in males, as shown in Table 5, whereas in females, associations with FS-H were stronger than with FS-D, as depicted in Table 6. Across both groups, FSI remained statistically insignificant in its association with skeletal or dental maturity indicators.

**Table 5:** Gender-wise Spearman’s correlation analysis of FS-H, FS-D, and FSI with calcification stages (CS) of permanent canine and second molar among males. p < 0.05 indicates statistical significance; p < 0.001 indicates high statistical significance. r = 0.20-0.399, low correlation; r = 0.40-0.599, moderate correlation; r = 0.60-0.799, high correlation. * Correlation is significant at the 0.05 level (two-tailed); ** Correlation is significant at the 0.01 level (two-tailed). Dental calcification stages were assessed using the method described by Demirjian et al. (1973) [1]. Frontal sinus dimensions were measured, and the frontal sinus index (FSI) was calculated based on the study by Alijani et al. (2020) [11], which was derived from the method described by Ertürk (1968) for frontal sinus measurements [14]. FS-H: frontal sinus height; FS-D: frontal sinus depth

CS of the tooth	Spearman’s correlation coefficient and p-value	FS-H (mm)	FS-D (mm)	FSI = FS-H/FS-D
CS 13	Correlation coefficient	0.410^*^	0.393^*^	0.003
	p-value	0.024	0.032	0.987
CS 23	Correlation coefficient	0.454^*^	0.461^*^	-0.080
	p-value	0.012	0.010	0.675
CS 33	Correlation coefficient	0.420^*^	0.444^*^	-0.095
	p-value	0.021	0.014	0.616
CS 43	Correlation coefficient	0.414^*^	0.390^*^	-0.055
	p-value	0.023	0.033	0.772
CS 17	Correlation coefficient	0.461^*^	0.493^**^	-0.145
	p-value	0.010	0.006	0.444
CS 27	Correlation coefficient	0.466^**^	0.510^**^	-0.147
	p-value	0.009	0.004	0.440
CS 37	Correlation coefficient	0.459^*^	0.474^**^	-0.112
	p-value	0.011	0.008	0.557
CS 47	Correlation coefficient	0.396^*^	0.452^*^	-0.148
	p-value	0.030	0.012	0.435

**Table 6:** Gender-wise Spearman’s correlation analysis of FS-H, FS-D, and FSI with calcification stages (CS) of permanent canine and second molar among females. p < 0.05 indicates statistical significance; p < 0.001 indicates high statistical significance. r = 0.20-0.399, low correlation; r = 0.40-0.599, moderate correlation; r = 0.60-0.799, high correlation. * Correlation is significant at the 0.05 level (two-tailed); ** Correlation is significant at the 0.01 level (two-tailed). Dental calcification stages were assessed using the method described by Demirjian et al. (1973) [1]. Frontal sinus dimensions were measured, and the frontal sinus index (FSI) was calculated based on the study by Alijani et al. (2020) [11], which was derived from the method described by Ertürk (1968) for frontal sinus measurements [14]. FS-H: frontal sinus height; FS-D: frontal sinus depth

CS of the tooth	Spearman’s correlation coefficient and p-value	FS-H (mm)	FS-D (mm)	FSI = FS-H/FS-D
CS 13	Correlation coefficient	0.516^**^	0.475^**^	0.136
	p-value	0.004	0.008	0.474
CS 23	Correlation coefficient	0.507^**^	0.451^*^	0.163
	p-value	0.004	0.012	0.390
CS 33	Correlation coefficient	0.486^**^	0.351	0.234
	p-value	0.007	0.057	0.212
CS 43	Correlation coefficient	0.542^**^	0.439^*^	0.221
	p-value	0.002	0.015	0.240
CS 17	Correlation coefficient	0.403^*^	0.347	0.162
	p-value	0.027	0.060	0.393
CS 27	Correlation coefficient	0.415^*^	0.342	0.180
	p-value	0.023	0.064	0.342
CS 37	Correlation coefficient	0.412^*^	0.337	0.188
	p-value	0.024	0.069	0.320
CS 47	Correlation coefficient	0.447^*^	0.310	0.241
	p-value	0.013	0.095	0.199

## Discussion

The integration of frontal sinus evaluation with established skeletal and dental maturity markers enhances the diagnostic value of conventional radiographs without requiring additional imaging or radiation exposure, unlike the study done by Uysal et al. [4], which used hand wrist radiographs along with LC. The stratified random sampling employed in this study ensured balanced representation across six CVMI stages and both genders, enhancing the robustness and applicability of the findings. The age range of eight to 20 years was specifically chosen as it encapsulates the critical window of pubertal growth. Previous studies have indicated that frontal sinus pneumatization becomes measurable around eight years and reaches peak development during adolescence, aligning closely with skeletal and craniofacial growth patterns [5,7]. Similarly, the calcification of permanent canines and second molars occurs within this age range, closely aligning with the pubertal growth spurt [1].

To evaluate skeletal maturity, the CVMI method developed by Baccetti et al. [13] was employed because it is more reproducible and objective compared to earlier cervical vertebrae maturation (CVM) models, providing a strong correlation with the timing of pubertal growth spurts. Dental maturity was assessed using DI, which classifies tooth development into eight calcification stages (A to H) based on radiographic appearance [1]. This approach, widely validated across populations, offers a stable marker of dental development unaffected by eruption patterns. Frontal sinus measurements, particularly height and depth, were obtained using the method described by Ertürk [14], which calculates an FSI as the ratio of height to width, thereby minimizing magnification errors and inter-individual variability.

FS-H and FS-D showed statistically significant differences across CVMI stages, reflecting an increase in sinus dimensions both vertically and anteroposteriorly with advancing skeletal maturity. However, the FSI remained stable, suggesting that while the sinus grows, its proportional dimensions remain consistent. Pairwise comparisons confirmed notable differences between early and late CVMI stages, particularly between stages I vs. VI and II vs. VI for FS-H, and between stages I, IV vs. V and VI for FS-D. These findings indicate that the frontal sinus shows growth between ages eight and 20, with pronounced growth apparent in the later stages of skeletal development of cervical vertebrae. Interestingly, the FSI did not exhibit significant differences across the CVMI stages (p > 0.05 in all comparisons), further validating that the sinus expands in both height and depth in a proportional manner. The lack of change in the FSI implies that while absolute growth occurs, its proportionality remains stable across developmental stages.

Spearman correlation analysis revealed a moderate to strong positive relationship between FS-H and CVMI (r = 0.550), and between FS-D and CVMI (r = 0.485), supporting the hypothesis that frontal sinus development parallels cervical vertebral maturation. The FSI, however, did not show significant correlation with CVMI stages (p = 0.973). Similar trends were observed in gender-based analyses, with stronger correlations noted among males. These findings further establish that frontal sinus development follows a pattern of progressive increase in size during skeletal growth of cervical vertebrae, making it a potential auxiliary indicator for skeletal maturity assessment, especially in males.

The findings of the current study correspond to the results of a study done by Alfawzan [12] in 2023, which stated that there was a significant correlation of FS-H and width with CVMI stages in both sexes, but the FSI was not significantly correlated to CVMI. However, the present study is in contrast with a study conducted by Alijani et al. [11] in 2020, which reported that the FSI and width were significantly greater in males, but the sinus length was not significantly different between males and females (P = 0.383). In this study, the mean FSI had a significant correlation with the cervical vertebrae maturity stage in both groups. In a study done by Mahmood et al. [10] in 2016, they found a significant association between frontal sinus height and width and cervical stages for both sexes, and changes in the FSI were significant in male subjects only.

In the combined analysis of all samples, a statistically significant moderate positive correlation was observed between the calcification stages of permanent canine and second molar for both FS-H and FS-D (p < 0.01), but the FSI did not show any significant correlation, as indicated by p-values > 0.05. This suggests that while the absolute size of the sinus increases with dental maturity, the proportional ratio of height to depth remains relatively unchanged. Among male subjects, significant positive correlations were observed between the calcification stages of all evaluated teeth and both FS-H and FS-D (p < 0.05 or 0.01). This indicates that the increase in dental calcification stages is associated with increased sinus dimensions. In the female subgroup, the calcification stages of permanent maxillary and mandibular canines and second molars exhibited significant positive correlations with FS-H (p < 0.05 or 0.01). In contrast, FS-D correlations were weaker and non-significant for many of the teeth. However, the FSI showed no significant correlation with dental calcification stages (p > 0.05) in males and females.

According to Chertkow in 1980, tooth mineralization was reported to be a reliable indication of pubertal growth spurt [3]. Several studies have explored the link between dental calcification stages and skeletal maturity, showing that certain teeth can be useful indicators of growth phases. Trakinienė et al. [15] in 2015 found strong correlations between cervical vertebral maturity and the calcification stages of the maxillary canine and mandibular second molar, suggesting these teeth can help identify key stages of growth. Similarly, Mehta et al. [16] in 2016 reported a strong association between DI and CVMI, and highlighted the mandibular third molar as a helpful additional tool for assessing skeletal maturity.

However, other researchers have noted certain limitations. Toodehzaeim et al. [17] in 2020 pointed out that while the mandibular second molar shows a high correlation with skeletal maturity, it may only be reliable for identifying post-pubertal growth stages, and not earlier phases. In a systematic review, Barreto et al. [18] in 2022 concluded that although there seems to be a positive correlation between dental and cervical vertebral maturation, the overall quality of available evidence is still considered low and requires further high-quality research.

To the best of our knowledge, this is the first study that brings together an analysis of frontal sinus development with the calcification stages of permanent teeth. While past research has often focused on either dental or skeletal maturity, few researchers have explored frontal sinus growth separately, and none have combined all three parameters within a single study framework. By doing so, this study offers a fresh perspective on assessing skeletal maturity, using markers that are already available on routine radiographs, without needing additional imaging or radiation exposure. Each skeletal maturity marker has limitations when used in isolation. In our integrated approach, not only adds depth to existing methods but also highlights the potential of using more accessible indicators in clinical orthodontic and pediatric assessments. It opens up new possibilities for refining age-estimation techniques and improving the timing of growth-related treatments.

This study had several inherent limitations. Being retrospective in design, it relied on existing radiographs, which restricted the ability to observe longitudinal changes within individuals over time. Although the sample size was adequate for initial analysis, a larger cohort would enhance statistical strength and generalizability. Additionally, frontal sinus morphology exhibits considerable individual variation, and in some subjects, underdevelopment or absence of the sinus may compromise its reliability as a growth marker. Since the study focused on a specific population, its findings may not be universally applicable without validation in other ethnic or geographic groups. Lastly, while the FSI was included to reduce individual variability, it showed weaker correlation with growth indicators compared to direct linear measurements, suggesting limited utility in isolation.

The findings of this study open avenues for broader research into the combined use of frontal sinus development, dental calcification, and skeletal maturity indicators in clinical diagnostics. Future studies could benefit from longitudinal designs that track individuals through different stages of growth to better capture developmental trends and strengthen causal inference. Expanding the sample size and including diverse populations would also improve the external validity of the results. Also, emerging technologies, including AI-driven radiograph interpretation and 3D imaging, are poised to enhance the precision of growth assessments. Integrating such tools with established radiographic indicators may lead to more personalized and accurate treatment planning.

## Conclusions

This study highlights the value of integrating frontal sinus dimensions, CVMI, and dental calcification stages as complementary indicators for skeletal maturity assessment. Moderate positive correlations were found between FS-H, FS-D, and CVMI stages, indicating their relevance in growth evaluation. However, the FSI did not show significant variation across CVMI stages, limiting its use as a proportional growth marker. Calcification stages of permanent canines and second molars also showed moderate correlations with FS-H and FS-D, reinforcing the sinus's potential role in maturity assessment. FSI, however, lacked meaningful association with dental maturity. Notably, correlations were slightly stronger in males than in females. These findings suggest that linear frontal sinus measurements serve as useful adjuncts in assessing skeletal maturity, whereas FSI may have limited clinical applicability. Overall, the integration of frontal sinus analysis with CVMI and dental calcification markers presents a moderately reliable, practical, non-invasive, and cost-effective approach to growth assessment, supporting improved orthodontic and pediatric treatment planning.
